# Anoxygenic phototrophic purple non-sulfur bacteria: tool for bioremediation of hazardous environmental pollutants

**DOI:** 10.1007/s11274-023-03729-7

**Published:** 2023-08-18

**Authors:** Kartik Dhar, Kadiyala Venkateswarlu, Mallavarapu Megharaj

**Affiliations:** 1 Global Centre for Environmental Remediation (GCER), College of Engineering, Science and Environment, University of Newcastle, Callaghan, NSW 2308 Australia; 2 Department of Microbiology, Faculty of Biological Sciences, University of Chittagong, Chittagong, 4331 Bangladesh; 3 Formerly Department of Microbiology, Sri Krishnadevaraya University, Anantapuramu, Andhra Pradesh 515003 India; 4 Cooperative Research Centre for Contamination Assessment and Remediation of the Environment (CRC CARE), University of Newcastle, Callaghan, NSW 2308 Australia

**Keywords:** Purple non-sulfur bacteria (PNSB), Bioremediation, Biodegradation, Heavy metal resistance, Hazardous organic pollutants

## Abstract

The extraordinary metabolic flexibility of anoxygenic phototrophic purple non-sulfur bacteria (PNSB) has been exploited in the development of various biotechnological applications, such as wastewater treatment, biohydrogen production, improvement of soil fertility and plant growth, and recovery of high-value compounds. These versatile microorganisms can also be employed for the efficient bioremediation of hazardous inorganic and organic pollutants from contaminated environments. Certain members of PNSB, especially strains of *Rhodobacter sphaeroides* and *Rhodopseudomonas palustris*, exhibit efficient remediation of several toxic and carcinogenic heavy metals and metalloids, such as arsenic, cadmium, chromium, and lead. PNSB are also known to utilize diverse biomass-derived lignocellulosic organic compounds and xenobiotics. Although biodegradation of some substituted aromatic compounds by PNSB has been established, available information on the involvement of PNSB in the biodegradation of toxic organic pollutants is limited. In this review, we present advancements in the field of PNSB-based bioremediation of heavy metals and organic pollutants. Furthermore, we highlight that the potential role of PNSB as a promising bioremediation tool remains largely unexplored. Thus, this review emphasizes the necessity of investing extensive research efforts in the development of PNSB-based bioremediation technology.

## Introduction

Purple non-sulfur bacteria (PNSB) are a group of diverse collections of anoxygenic photosynthetic microorganisms belonging to the α- and β-proteobacterial classes. They are anoxygenic phototrophs: unable to use water as the electron donor during photosynthesis, and hence, do not evolve oxygen during the process (Madigan and Jung 2009). PNSB are regarded as the most metabolically versatile among bacteria because of their ability to adapt to almost all known nutritional modes. Depending on the environmental conditions, they can grow as photoautotrophs, photoheterotrophs, chemolithoautotrophs, or chemoorganotrophs.

**Fig. 1:**
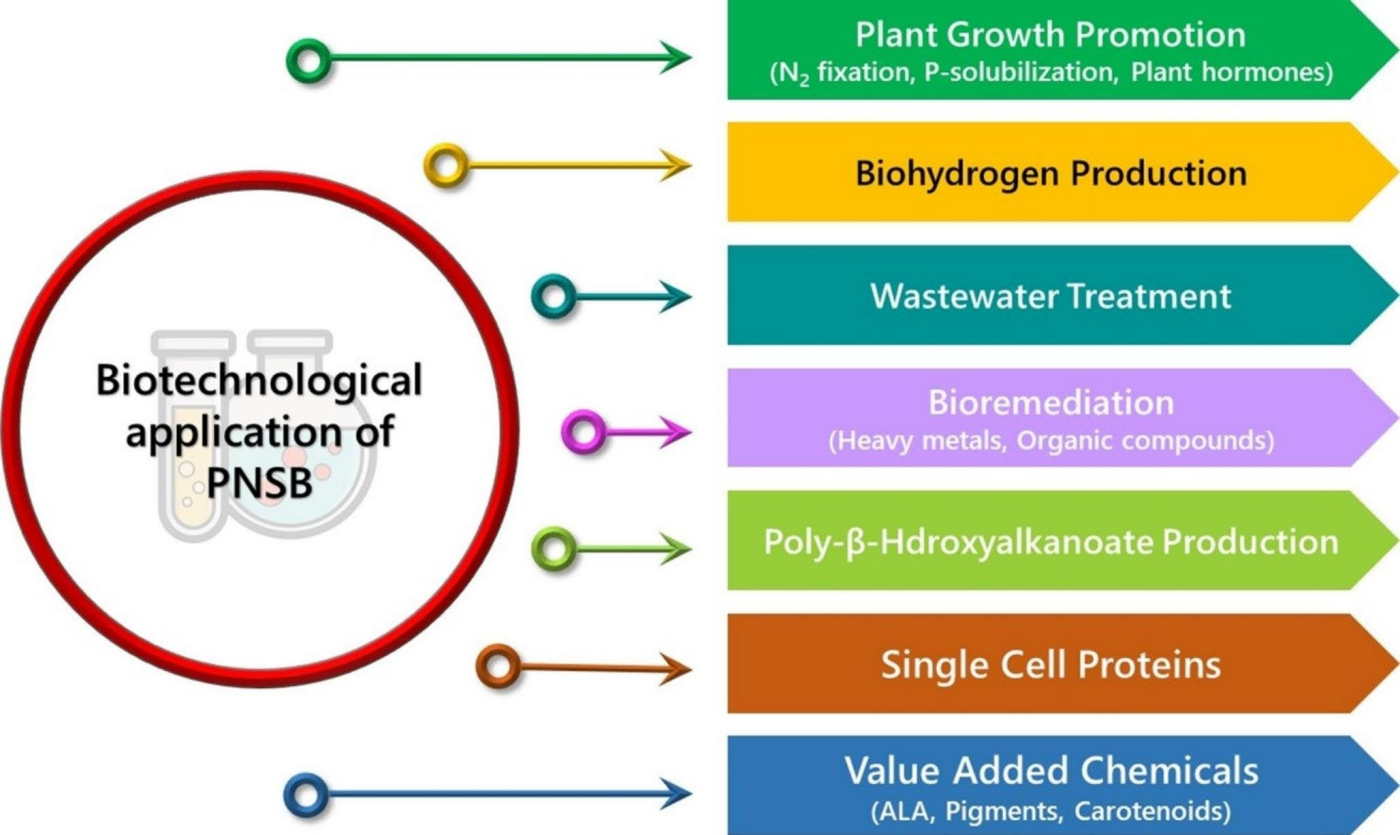
Biotechnological applications of purple non-sulfur bacteria (PNSB). PNSB are extensively exploited in wastewater treatment technology and biohydrogen production. PNSB-based wastewater treatment process is also linked with the recovery of high-value products, such as poly-β-hydroxyalkanoates, carotenoid pigments, 5-aminolevulinic acid (ALA), and single-cell proteins. In addition, PNSB exhibit plant growth-promoting traits. Remarkably, PNSB are promising agents for the bioremediation of toxic heavy metals and hazardous organic pollutants

The metabolic flexibility of PNSB serves as the basis for their extensive utilization in biotechnology. Over the last few decades, PNSB have therefore been instrumental in the development of several biotechnological applications (Fig. 1). PNSB-based wastewater treatment and biohydrogen production have received significant research interest (Kim et al. 2004; Tao et al. 2008; Tiang et al. 2020). Wastewater treatment employing PNSB is considered a sustainable, eco-friendly, and cost-effective technology (Cao et al. 2020; Chen et al. 2020). Additionally, the recovery of valuable resources such as poly-β-hydroxyalkanoates, single-cell proteins, pigments and carotenoids from the PNSB-mediated wastewater treatment process contributes to the circular economy (Lu et al. 2019; Cao et al. 2020; Sharma et al. 2020; Wada et al. 2022). PNSB are well known for their capabilities of plant growth promotion by nitrogen fixation, phosphate solubilization, and indole acetic acid production (Koh and Song 2007; Sakpirom et al. 2017; Maeda 2021).

Environmental pollution is a global issue that poses a serious threat to all living organisms, including humans. Since the rapid expansion of the industrial revolution, anthropogenic sources have exceeded emitting pollutants compared to natural sources. Extensive industrial activities, inappropriate waste disposal, indiscriminate exploitation of the natural environment, massive-scale urbanization, and rapid growth of world population exacerbate the quality of the natural environment by introducing an enormous amount of organic and inorganic pollutants (Liang et al. 2019; Liang and Yang 2019; Ukaogo et al. 2020). Microorganisms are well known for their ability to degrade or detoxify a variety of pollutants and restoration of the ecosystem. Numerous bacteria, fungi, actinomycetes, and algae belonging to different phylogenetic and functional groups capable of bioremediation of hazardous substances have been described in the literature (Leahy and Colwell 1990; Fuchs et al. 2011; Verma and Kuila 2019). Simultaneously, genetic and biochemical mechanisms of detoxification processes have been elucidated, and the beneficial microorganisms or their genes or metabolic products have been exploited for the bioremediation of contaminated environments. In recent times, the power of next-generation “omics” technologies and system biology approaches are being exploited to unveil the extraordinary bioremediation potentials of microorganisms (de Lorenzo 2008; Desai et al. 2010; Paliwal et al. 2012; Chandran et al. 2020).

The potential of PNSB in bioremediation has been known for decades. Many members reported in the literature are quite resistant to several toxic heavy metals or metalloids, while some others are capable of recalcitrant organic pollutant degradation. There are many excellent reviews available on PNSB-based wastewater treatment (Lu et al. 2019; Chen et al. 2020), biohydrogen production (Ghosh et al. 2017; Tiang et al. 2020), resource recovery (Capson-Tojo et al. 2020; George et al. 2020) and sustainable agriculture (Sakarika et al. 2020). Although some of the reviews mentioned the role of PNSB as a bioremediation tool, a critical appraisal of the literature evaluating the progress in the field is lacking. In this review, we summarize the noteworthy achievements in studies that dealt with the capabilities of PNSB in the bioremediation of heavy metals and metalloids, and hazardous organic environmental pollutants. We also provide a critical appraisal of the available literature and evaluate the suitability of PNSB in potential bioremediation applications. Based on the current research achievements and trends in the field, recommendations for future research are offered. Since previous reviews complied with the contributions of PNSB in wastewater treatment, including those containing lignocellulosic waste, biodegradation of non-hazardous organic compounds by PNSB is excluded from the review.

## Habitats of PNSB

PNSB are widely distributed in aquatic and terrestrial habitats; sunlight exposed, water-logged, oxygen-deficient microaerobic to anaerobic environments are ideal for their proliferation (Imhoff 2017). They have frequently been isolated from a variety of natural environments such as freshwater ponds, lakes, mud sediment, paddy field, marine water, and coastal sediment. Besides, PNSB can occupy a range of manmade polluted environments, as found in sewage sludges and wastewater ditches (Madigan and Jung 2009). In the natural environment, PNSB co-exists with other phototrophic bacteria. Sulfide concentration has a notable influence on the presence and abundance of PNSB. A high sulfidic environment with high-strength organic matter often favours the proliferation of purple sulfur bacteria that results in the formation of massive blooms and mats (Imhoff 2017). In contrast, PNSB grows well in low-sulfide, sunlight-exposed, organic-rich microaerobic or anaerobic environments. Unlike phototrophic purple sulfur bacteria, PNSB rarely forms massive blooms or microbial mats. In a swine wastewater ditch that contained acetate and propionate as the major organic carbon sources, members of the genera *Rhodobacter* and *Rhodopseudomonas* were found as the dominant inhabitants among phototrophic bacteria (Okubo et al. 2006). In another atypical case, several PNSB members were found dominating the microbial community of red-pink blooms and mats in wastewater ditches (Hiraishi et al. 2020). The massive proliferation of PNSB was suggested to be dependent on low dissolved oxygen levels, adequate light, low sulfide concentration, and the presence of high-strength organic matter (Okubo et al. 2006; Hiraishi et al. 2020). Some members of PNSB have been isolated from extreme environments. *Blastochloris tepida* (Favinger et al. 1989; Madigan et al. 2019), *Rhodoplanes tepidicaenis* and *Rhodoplanes azumiensis* (Hiraishi 2017) have been described as thermotolerant PNSB. Alkaliphilic *Rhodobaca bogoriensis* (Milford et al. 2000) *and Rhodobaca barguzinensis* (Boldareva et al. 2008) were isolated from soda lakes. *Rhodovastum atsumiense* isolated from submerged paddy soil (Salama et al. 2020) and *Rhodoblastus sphagnicola* isolated from *Sphagnum* peat bog (Kulichevskaya et al. 2006) have been described as acidophilic PNSB.

## Physiology of PNSB

**Fig. 2:**
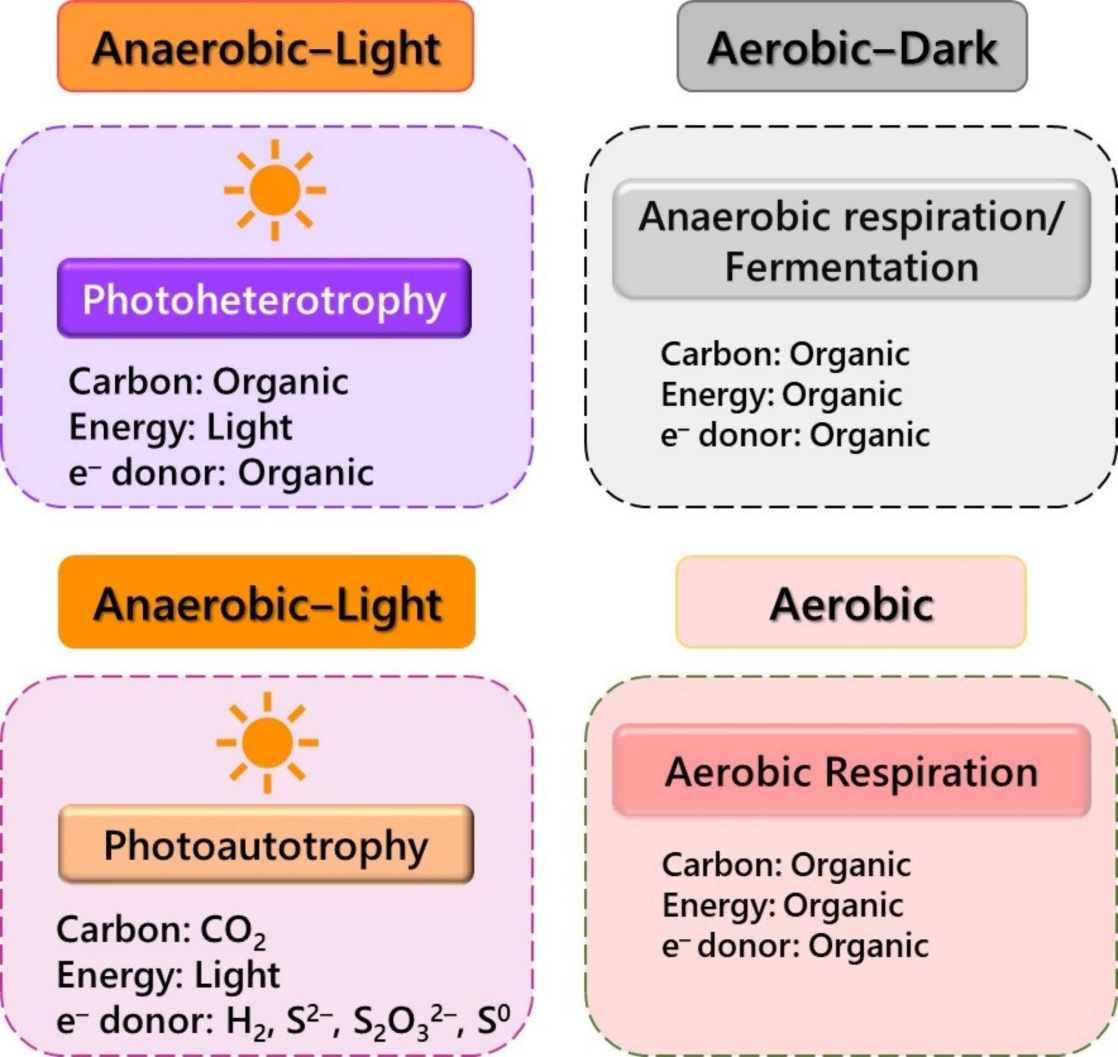
Metabolic flexibility of PNSB in adapting different modes of growth. They can grow as photoautotrophs by fixing CO_2_ in anoxygenic photosynthesis under anaerobic-light conditions with a reduced electron donor other than H_2_O. Photoheterotrophy is the principal mode of growth under anaerobic-light conditions with organic substrates. In the dark, they can grow by fermentation or anaerobic respiration. Chemoheterotrophic growth on organic substrates in an aerated environment is also possible in PNSB.

PNSB can grow under a variety of environmental conditions and exhibit great flexibility in adopting different nutrition modes (Fig. 2). They can grow in anaerobic, microaerobic and aerobic conditions, and in the presence or absence of light. Under anaerobic-light conditions, they grow photoautotrophically by fixing CO_2_ or photoheterotrophically using a variety of organic compounds as electron donors. PNSB are anoxygenic photoautotrophs capable of light-dependent bacteriochlorophyll-mediated CO_2_ fixation and phototrophic energy transfer. Bacteriochlorophyll *a* or *b* and carotenoids are the major pigments in PNSB (Imhoff et al. 2005). Their pigment production and photosynthetic apparatus formation are sensitive to oxygen and occur only under anaerobic or microaerobic light conditions. During photosynthesis, they cannot use H_2_O as an electron donor, but rather utilize other reduced chemicals. Molecular H_2_ or low concentration of HS^–^ serves as the typical electron donor for photoautotrophic growth. However, S_2_O_3_^2–^ and Fe^2+^ can sometimes be used as electron donors. In the presence of high-strength organic matter under microaerobic/anaerobic-light conditions, PNSB grow preferentially as photoheterotrophs. They can utilize a wide variety of organic compounds; several organic acids, fatty acids, alcohols, carbohydrates, and amino acids are utilized for photoheterotrophic growth. They grow best with organic acids, such as malate, succinate, acetate, and pyruvate. Some species can also grow on lactate or propionate. In addition, certain members can utilize benzoates, hydroxybenzoate, chlorobenzoates, toluene, polyacrylamide, hydrocarbons, and pesticides (Imhoff et al. 2005; Madigan and Jung 2009).

In the absence of light, PNSB can switch to anaerobic dark respiration or fermentation or aerobic respiration. Certain PNSB can obtain energy by fermenting sugars or electron-transport coupled anaerobic respiration under anaerobic dark conditions (Imhoff 2008; Madigan and Jung 2009). In addition, PNSB can grow aerobically by deriving carbon from organic compounds and energy from chemical molecules. Although certain species of PNSB are sensitive to oxygen, many of them grow vigorously in aerated environments. Organic acids, which can be utilized for photoheterotrophic growth, serve as the carbon source and electron donor for aerobic dark respiration, with O_2_ as the terminal electron acceptor (Madigan and Jung 2009).

## Advantages of PNSB as bioremediation agents

**Fig. 3:**
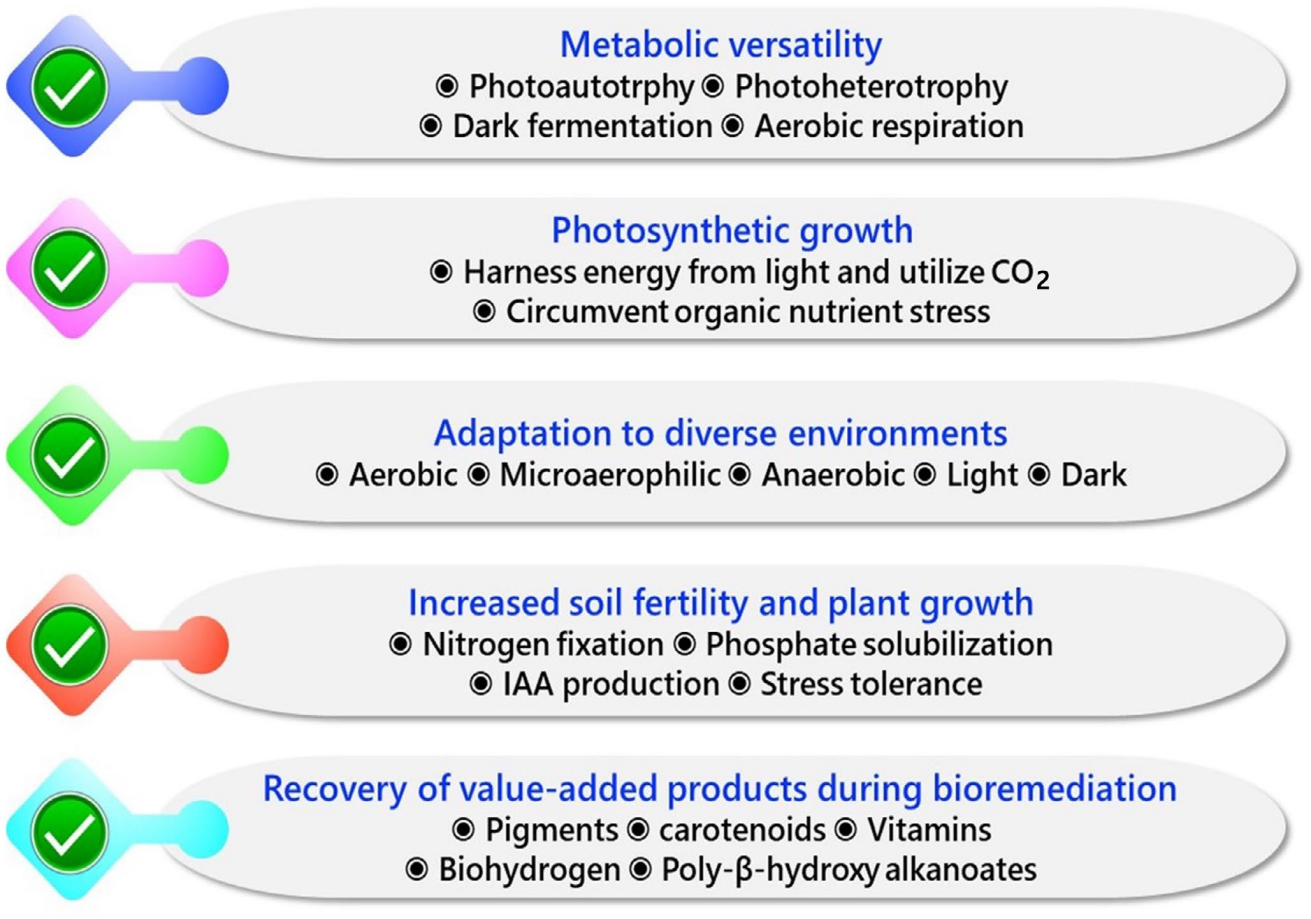
Prominent characteristics of PNSB that would be advantageous in efficient bioremediation of environmental pollutants along with other benefits including increased plant growth and recovery of value-added products

The major advantages of using PNSB as bioremediation agents are summarized in Fig. 3. PNSB can switch to different growth modes depending on their environment. The limitation of organic nutrients and the absence of an appropriate electron acceptor often limit the growth and function of bacteria employed in areas contaminated with pollutants (Megharaj et al. 2011). For instance, heavy metal-contaminated wastewater or soil with low organic carbon might fall short in supporting the growth of metal-bioremediating heterotrophic bacteria. Since PNSB can grow photoautotrophically under microaerobic/anaerobic light conditions, they should have the capability to overcome organic nutrient limitations. Also, unlike strict anaerobic sulfate-reducing or methanogenic anaerobic bacteria, some PNSB are quite tolerant to oxygen. Additionally, the ability to utilize diverse organic carbon sources, especially cellulosic residues, carbohydrates, and organic acids, would be beneficial in promoting the growth of PNSB that bioremediate contaminated environments. Shallow water bodies, such as ponds, paddy fields, eutrophication lakes, and coastal sediments, display seasonal or diurnal changes in light intensity and dissolved oxygen concentrations (Martin et al. 2013). The flexibility of PNSB toward aerobic-microaerobic/anaerobic and light-dark conditions could withstand such switches in natural environments. Many agricultural soils contaminated with toxic chemicals also suffer from nutrient limitations. Most of the PNSB can fix atmospheric nitrogen to NH_4_^+^ that can be used by plants (Sakpirom et al. 2017; Maeda 2021). PNSB have been reported to produce plant growth-promoting chemicals like indole-3-acetic acid (IAA) and 5-aminolevulinic acid (ALA) (Sakpirom et al. 2017) and solubilize insoluble phosphate (Koh and Song 2007) to available form. All these traits would be beneficial in increasing soil health and plant productivity in the contaminated soils to be remediated.

### Bioremediation of heavy metals and metalloids

Environmental contamination by heavy metals and metalloids is a serious ecological and public health concern. Heavy metals and metalloids are naturally occurring elements; however, extensive exploitation in various industrial, pharmaceutical, agricultural, and domestic applications are the major cause of their widespread occurrence in nature, often in concentrations high enough to manifest adverse health effects. Several metals and metalloids, such as arsenic (As), cadmium (Cd), mercury (Hg), lead (Pb), chromium (Cr), and several oxyanions like selenite and tellurite, possess the ability to cause serious diseases in different organs. Moreover, certain elements (e.g., arsenic, chromium, and cadmium) are mutagenic and carcinogenic (Tchounwou et al. 2012). Removal of heavy metals and metalloids is of paramount importance from environmental and human health perspectives. Microorganisms, especially bacteria and fungi, can immobilize or transform the toxic form of heavy metals and metalloids into non-toxic forms. Bacterial bioremediation of heavy metals and metalloids is an effective technology for the removal, detoxification, and restoration of contaminated environments (Verma and Kuila 2019). Like other heterotrophic bacteria, PNSB shows resistance to several toxic heavy metals and metalloids. Table 1 summarizes the available reports that describe heavy metal resistant PNSB, their mechanisms of resistance and intended applications.

**Table 1:** Purple non-sulfur bacteria resistant toward metals and metalloids, mechanisms of resistance, and their potential applications

Organism	Metal/ Metalloid	Resistance mechanism	Potential application	References
*Rhodopseudomonas palustris* strain C1 *R. palustris* strain AB3	As(III) AS(V)	Biofilm formation Redox transformation Glutathione production Methylcobalamine production Biomethylation and volatilization	As removal from contaminated paddy fields surrounding mine sites	Nookongbut et al. (2016)
*R. palustris* strain L28		Biofilm formation Redox transformation Glutathione production Methylcobalamine production		
*Rubrivivax benzoatilyticus* strain C31		Redox transformation Glutathione production Methylcobalamine production		
*R. palustris* strain CS2	As(V)	As(V) reduction	As removal and plant growth promotion	Batool and Rehman (2017)
*Rhodopseudomonas faecalis* strain SS5	As(III)	As(III) oxidation		
*Rhodobacter* sp. strain BT18	As(III)	Complexation with exopolysaccharide (EPS)	As chelation from contaminated water	Govarthanan et al. (2019)
*Rb. sphaeroides*	Cd^2+^	Bioprecipitation as CdS	Cd removal	Bai et al. (2009)
*Rb. sphaeroides*	Cd^2+^	Immobilization by speciation change	Cd and/or Zn removal from contaminated soil; decreased phytoavailablity of Cd; enhanced Zn/Cd ratio in plant	Fan et al. (2012) Peng et al. (2018)
*Rubrivivax gelatinosus* strain TN414	Cd^2+^	NA	Cd and Zn removal from contaminated soil, plant growth promotion	Sakpirom et al. (2017)
*R. palustris* strain TN110	Cd^2+^	Bioprecipitation as CdS	Cd and Zn removal from contaminated soil, plant growth promotion	Sakpirom et al. (2017) Sakpirom et al. (2019)
*Rb. sphaeroides* strain S *Rhodovulum* sp. strain PS88	Cd^2+^	Biosorption	Cd removal	Watanabe et al. (2003)
*Rhodobium marinum* strain NW16	Cd^2+^	Complexation with EPS and bioaccumulation	Heavy metal removal from shrimp ponds	Panwichian et al. (2010a) Panwichian et al. (2010b) Panwichian et al. (2011)
	Pb^2+^			
	Zn^2+^			
	Cu^2+^			
*Rhodobacter sphaeroides* strain KMS24	Cd^2+^	Complexation with EPS and bioaccumulation	Heavy metal removal from shrimp ponds	Panwichian et al. (2010a) Panwichian et al. (2010b) Panwichian et al. (2011)
	Pb^2+^			
	Zn^2+^			
	Cu^2+^			
*Rhodobacter capsulatus*	Zn^2+^	Biosorption	Preparation of Zn biosorbent	Magnin et al. (2014)
*Rb. sphaeroides*	Zn^2+^	Bioprecipitation of ZnS	ZnS semiconductor	Bai et al. (2006)
*Rb. sphaeroides*	Pb^2+^	Bioprecipitation as PbSO_4_ and PbS	Pb removal from soil and reduction of Pb phytoavailability	Li et al. (2016)
*Rb. spharoides* strain 26	Cr(VI)	Intracellular chromate reduction	Chromate removal	Buccolieri et al. (2006)
*R. sphaeroides* strain 158	Cr(VI)	Intracellular reduction by chromate reductase	Chromate removal from liquid waste	Nepple et al. (2000)
*Rhodobacter* sp. strain GSKRLMBKU–03	Cr(VI)	Chromate reduction	Chromate removal from contaminated water	Rajyalaxmi et al. (2019)
*Afifella marina* strain SSS2-1	Hg^2+^	Volatilization to Hg^0^ Biosorption by live and dead cells	Mercury removal from contaminated shrimp pondss	Mukkata et al. (2015) Mukkata et al. (2019)
*Afifella marina* strain SSW15-1 *Rhodovulum sulfidophilum* strain SRW1-5	Hg^2+^	Volatilization to Hg^0^	Mercury removal from contaminated shrimp pondss	Mukkata et al. (2015)
*Rhodobacter* sp. strain NKPB030619	Selenite	Reduction to Se^0^	Selenite removal from marine environment	Yamada and Matsunaga 1997
*Rhodospirillum rubrum*	Selenite	Reduction to Se^0^	NA	Kessi et al. (1999)
*Rb. sphaeroides*	Tellurite	Reduction to Te^0^	Tellurite reduction	Moore and Kaplan (1992)
*Rb. sphaeroides* strain 2.4.1	Tellurite	Reduction to Te^0^	Tellurite reduction	O’Gara et al. (1997)
*Rhodopseudomonas palustris*	U	Reduction of U(VI) to U(IV) phosphate or carboxylate	U removal from contaminated soil and wastewater	Llorens et al. (2012)
*Rb. sphaeroides* strain SSI	Radioactiove Cs	Biosorption on EPS and active uptake	Radioactive Cs removal from soil, sediment mud and water	Sasaki et al. (2012a) Sasaki et al. (2012b)

NA, Not available

### Arsenic

Contamination of soil and water with the highly toxic and carcinogenic element As is a major environmental concern (Chung et al. 2014; Podgorski and Berg 2020). Exposure to As can cause the development of cancer in lungs, skin, kidneys, liver, and bladder. It is prevalent in drinking and cooking water, grains and vegetables, and seafood in different countries in the world. Arsenite [As(III)] is generally more toxic than arsenate [As(V)] and organic As compounds (Mandal and Suzuki 2002; Abernathy et al. 2003; Yang and Rosen 2016; Verma and Kuila 2019). Bacterial remediation of As has been investigated for decades, and several mechanisms such as redox transformation, biomethylation, and volatilization confer resistance to this toxic chemical (Yang and Rosen 2016; Ben Fekih et al. 2018).

Certain As-resistant strains of PNSB have been reported. Nookongbut et al. (2016) isolated four As-resistant PNSB from soil and water samples taken from polluted mine sites. Three strains of *Rhodopseudomonas palustris* (AB3, C1, and L28) and *Rubrivivax benzoatilyticus* strain C31 exhibited resistance to As(III) and As(V) under both anaerobic-light and aerobic-dark conditions in the order of C1 > AB3 > C31 > L28. In the supernatant of C1 culture grown under microaerobic-light conditions, strain C1 produced arsenobetaine with As(III) and monomethylarsonic [MMA(V)] acid with As(V). Moreover, volatile methylated As species such as dimethylarsenic acid [DMA(V)] and MMA(V) were detected with As(III) and As(V). Later, Nookongbut et al. (2017) provided evidence for the presence of several As marker genes, including arsenite transporter (*acr*3), arsenite oxidation (*aio*A), arsenate reduction (*ars*C), and As methylation (*ars*M) in strain C1. Moreover, strain C1 could perform both As(III) oxidation and As(V) reduction under aerobic-dark and anaerobic-light conditions, respectively. These results suggest that certain PNSB have the genetic makeup to cope with As contamination and the presence of multiple mechanisms for detoxification can serve as the basis for efficient removal of the contaminant from the impacted environment. *R. palustris* strain C1 and *R. benzoatilyticus* strain C31 were applied to alleviate toxicity from soil contaminated with 30 mg L^–1^ each of As(III) and As(V). When inoculated as a mixture at 1:1 ratio, there was a significant reduction of toxicity, as evident from the improved rice seed germination index. Moreover, the mixed culture produced ALA, IAA, exopolymeric substances, and siderophores that enhanced plant production and reduced As accumulation in rice (Nookongbut et al. 2018).

Exopolysaccharide (EPS) production and complexation with As is another mechanism of As-resistance in bacteria. So far, few EPS-producing As-resistant PNSB strains have been reported. For instance, EPS produced by *Rhodobacter* (*Rb.*) sp. BT18 had the capabilities of complexation with As and Pb (Govarthanan et al. 2019). The amount of EPS production was enhanced by culturing under anaerobic conditions with blue light. The extracted EPS at 1% concentration was efficient in the removal of 64% of the initial 100 mg L^–1^ As from the aqueous solution. These findings suggested that the EPS extract would be an attractive alternative for removing As from wastewater. Panwichian et al. (2011) isolated two EPS-producing PNSB, *Rhodobium marinum* NW16 and *Rb. sphaeroides* KMS24, from heavy metal-contaminated shrimp pond water. The extracted EPS in solutions were more effective than the whole cells of the bacteria suggesting their potential to remove heavy metals from contaminated waterbodies.

Several genes for As detoxification have been detected in certain strains of *R. palustris.* Some of those genes were introduced into other heterotrophic bacteria and even into rice. The engineered organisms developed resistance to As. The gene *arsM* for As(III)-S-adenosylmethyltransferase (AsrM) that catalyzes As(III) methylation was found in the genome of *R. palustris* CGA009. The expression of *arsM* gene, when introduced into As(III) sensitive strain of *E. coli*, resulted in the production of volatile As species and conferred resistance to As. The activity of the AsrM resulted in the formation of As(III) to less toxic methylated pentavalent species, dimethylarsinic acid [DMA(V)], trimethylarsine oxide (TMAO), and trimethylarsine [TMA(III)] gas (Qin et al. 2006). The *asrM* gene was also inserted into the chromosome of *Pseudomonas putida* KT2440. In this case also, methylation and subsequent detoxification of As(III) was evident from the formation of less toxic and volatile As species, such as MMA(V), DMA(V), TMAO and TMA(III) (Chen et al. 2014). As regulatory protein (ArsR) has high affinity and specificity to As(III). Genes for ArsR from *R. palustris* CGA009 were engineered into *E. coli* cells (Ke et al. 2018). The engineered cells harboring *arsR*_RP2_ exhibited the highest As adsorption capacity within a wider pH (5.0 ~ 9.0) and salinity (0 ~ 15.0 g L^–1^ NaCl) range. About 2.32 mg g^–1^ As(III) and 1.47 mg g^–1^ As(V) were adsorbed by *E. coli* (*arsR*_*RP2*_), and these amounts were 4.2- and 1.3-fold higher than the control strain, respectively. Meng et al. (2011) introduced the *asrM* gene into Japonica rice *Oryza sativa* cv *Nipponbare* and demonstrated the acquisition of As methylation trait in the transgenic plant. Methylated products, MMA(V) and DMA(V), were detected in the roots and shoots of transgenic rice. After 12 days of exposure, As loss from volatilization was significant. These studies suggest that As-resistant PNSB strains and secreted EPS can be utilized for the bioremediation of contaminated soil and water. Moreover, results from the studies with As-resistant transgenes in heterotrophic bacteria and plants encourage further development and testing of the recombinant gene technology in As bioremediation.

### Chromium

Cr, especially its hexavalent form Cr(VI), is highly toxic and carcinogenic. Contamination of water and soil with Cr(VI) is of serious environmental concern due to the adverse effects on plants, animals, and humans (Saha et al. 2011; DesMarias and Costa 2019). Bacterial reduction of highly toxic and water-soluble Cr(VI) to less toxic Cr(III) has been considered a major detoxification mechanism (Wang and Shen 1995; Pushkar et al. 2021; Rahman et al. 2023). However, only a few Cr(VI)-resistant PNSB have been documented so far. Buccolieri et al. (2006) suggested that the carotenoid-less mutant of *Rb. spharoides* strain 26 was tolerant to Cr(VI). Although CrO_4_^2–^ affected the morphology and caused a long lag in the anaerobically grown cells of the strain, the cells at stationary phase could reduce 0.20 mM CrO_4_^2–^ within 80–90 h (Italiano et al. 2012). Chromate reduction was mainly found in the cytoplasmic fraction suggesting that the CrO_4_^2–^ was initially transported inside the cells and later reduced to Cr(III) by cytoplasmic reductase activity. Another strain *Rb. sphaeroides* 158 (ATCC 17,023) could reduce up to 43 µM Cr(VI) to Cr(III) under both anaerobic-light and aerobic-dark conditions. The NADH-dependant chromate-reducing enzyme extracted from cells grown under anaerobic light was found to be constitutively produced suggesting that the enzyme production was independent of the presence of chromate. The reduction process was intracellular, mainly in the cytoplasm, while the reduced Cr(III) was found in the extracellular medium, indicating an active excretion of the product out of the cell (Nepple et al. 2000). Chromate reduction was also reported in another *Rhodobacter* sp. Free and alginate immobilized cells of the strain GSKRLMBKU–03 could also carry out the reduction of Cr(VI) to Cr(III) (Rajyalaxmi et al. 2019). Reduction of Cr(VI) to Cr(III) is a widespread metabolic feature of many aerobic and anaerobic bacteria inhabiting soil and sediment (Bopp and Ehrlich 1988; Turick et al. 1996; Pal and Paul 2004; Das et al. 2021). Yet, Cr(VI) resistance in PNSB remains relatively unknown.

### Cadmium

Cd is a non-essential highly toxic metal that can cause cancer in the breast, lung, prostate, pancreas, and kidneys (Genchi et al. 2020). Certain bacteria, fungi and algae can efficiently remove Cd from contaminated environments (Kumar et al. 2021). Some PNSB show resistance to Cd and their application in contaminated soils reduced the phytoavailabilty and bioaccumulation of the toxic element in plants. Watanabe et al. (2003) reported Cd biosorption by *Rb. sphaeroides* S and *Rhodovulum* sp. PS88. Interestingly, Cd removal was higher under aerobic-dark conditions than that found under anaerobic-light conditions. Another strain of *Rb. sphaeroides* could remove Cd from the culture medium. The strain could completely remove 40 mg L^–1^ Cd after 42 h of incubation. Biosorption of most of the Cd occurred when the cells reached their stationary phase. The Cd removal mechanism appeared to be the precipitation of the metal sulfide by the enzyme cysteine desulfhydrase. The activity of the enzyme was found higher at lower Cd concentrations at 10 and 20 mg L^–1^ Cd compared to that found at higher 30 and 40 mg L^–1^ Cd (Bai et al. 2008). Although cysteine desulfhydrase is a cytoplasmic enzyme and CdS particles were expected to be found in the cytoplasm, the authors found small amounts of the metal sulfide in the cytoplasm and membrane, while most of the precipitates were found on the cell wall. A similar mechanism of Cd removal through CdS precipitation was also reported in a *R. palustris* strain (Bai et al. 2009). However, unlike *Rhodobacter sphaeroides* (Bai et al. 2008), CdS biosynthesis in *R. palustris* was found in the cytoplasm and later the precipitates were excreted out of the cells (Bai et al. 2009).

A metal resistant *Rhodobacter sphaeroides* strain was isolated by Fan et al. (2012) from oil field injection water. The strain was investigated for the bioremediation of soil contaminated with Cd, soil co-contaminated with Cd and Zn (Peng et al. 2018), and wastewater contaminated with Cd and Zn (Li et al. 2017). Inoculation of the bacterium in Cd-contaminated soil decreased the phytoavailability by changing the speciation of Cd from exchangeable phases to more stable forms. The exchangeable phases of Cd decreased by 27–46% while the less accessible bound phase to Fe–Mn oxides increased by 22–44% in inoculated soils. Consequently, Cd accumulation decreased in wheat roots and leaves. Similar results were also observed when the strain was inoculated into soil co-contaminated with Cd and Zn (Peng et al. 2018). The soil inoculation reduced the exchangeable phases of both Cd and Zn in soil by 30.70 and 100%, respectively. Consequently, the Cd levels in leaf and root were reduced by 62.30 and 47.20%, respectively. However, at high concentrations of Cd and Zn, the bioremediation was inefficient. The same *Rb. sphaeroides* strain was used to remediate a simulated wastewater contaminated with 100 mg L^–1^ Zn and 50 mg L^–1^ Cd. The removal rates for Cd and Zn reached 97.92 and 97.76%, respectively. During the bioremediation process, the elements were removed through biosorption and precipitation (Li et al. 2017).

Certain strains of PNSB have been described for their ability for bioremediation of Cd and promotion of plant growth. Sakpirom et al. (2017) obtained *R. palustris* strain TN110 and *Rubrivivax gelatinosus* strain TN414 from Cd- and Zn-contaminated paddy fields. When grown in a liquid medium with 23.00 mg L^–1^ of Cd or 262.40 mg L^–1^ of Zn, the strain TN110 removed 84% and 55% removal of Cd and Zn after 48 h of incubation under microaerobic-light conditions. Strain TN414 removed 72% Cd and 74% Zn, respectively, under the same experimental conditions. In addition to Cd- and Zn-removing abilities, both strains exhibited several plant growth-promoting traits including indole acetic acid and 5-aminolevulinic acid production, and nitrogen fixation. The mechanism of Cd-resistance in the TN110 strain was the bioprecipitation of CdS nanoparticles by the action of cysteine desulfhydrase (Sakpirom et al. 2019). The synthesized CdS particles were mostly extracellular, and a minor fraction remained inside the cell. The CdS nanoparticles were less toxic to the cell having an IC50 value of 1.76 mM compared to Cd (II) which had an IC50 of 0.82 mM. Interestingly, the CdS nanoparticles at the IC50 value up-regulated the Mo-Fe nitrogenase gene (*nif*H) and V-Fe nitrogenase gene (*vnf*G), and down-regulated Fe-Fe nitrogenase gene (*anf*G). Due to the lower toxicity of CdS, precipitation of Cd by the strain would be a viable option for the remediation of contaminated soil with low levels of Cd. Additionally, Cd bioremediation by the organism could also enhance soil fertility and plant growth resulting from the promotion of biological nitrogen fixation.

### Lead

A Pb-resistant strain *Rhodobacter sphaeroides* SC01 was isolated from saline paddy soil. This bacterium could remove 98% of the initial 160 mg L^-1^ Pb^2+^ from a liquid medium. The authors suggested that the mechanism of Pb removal was biosorption and precipitation of lead phosphate hydroxide on the cell surface (Su et al. 2020). Another strain of *Rhodobacter sphaeroides*, which was isolated from oil field injection water (Fan et al. 2012), showed Pb resistance (Li et al. 2016). During bioremediation of Pb-contaminated soil, inoculation of the strain resulted in the decrease of more accessible and exchangeable phase and simultaneous increase of the less accessible residual fraction. Immobilization of Pb in soil also resulted in the decrease of phytoavailability of Pb. Precipitation of Pb as PbSO_4_ and PbS has been suggested as the main mechanism of Pb transformation (Li et al. 2016). The activity of cysteine desulfhydrase was also detected as a Pb resistance mechanism in *Rb. sphaeroides* (Bai and Zhang 2009). The bacterium produced cysteine desulfhydrase (C-S-lyase) that catalyzed the reaction of extracellular PbS nanoparticles. Heat-killed free cells or immobilized floc of *Rb. sphaeroides* were also utilized for the biosorption of Pb from an aqueous solution (Seki et al. 1998; Seki and Suzuki 2002). The metal biosorption mechanism was suggested as the binding of bivalent metal ion to the carboxylic and phosphatic-type sites on the bacterial cell surface.

### Selenite

A marine PNS bacterium, *Rhodobacter* sp. strain NKPB030619, was isolated by Yamada et al. (1997b) that could tolerate over 5 mM selenite. Within five days of incubation, the strain reduced almost 99% of the added 1.10 mM selenite to elemental selenium. Selenite reduction was dependent on the incubation conditions: an anaerobic-light condition with malic acid supplementation in the medium resulted in a far significant amount of selenite reduction, while an anaerobic-dark condition and the absence of malate severely limited the process. Selenite resistance was also described in *Rhodospirillum rubrum* which could completely reduce 1.50 mM selenite to elemental selenium (Kessi et al. 1999). Extensive selenite reduction was evident under anaerobic-light conditions while incubation under aerobic condition was inefficient in inducing appreciable selenite transformation. Two mechanisms have so far been suggested for phototrophic selenite reduction. The accumulation of elemental selenium in the culture supernatant of *Rhodobacter* sp. strain NKPB030619 suggested that extracellular reduction was the main mechanism involved (Yamada et al. 1997b). In contrast, Kessi et al. (1999) demonstrated that selenite reduction in *Rhodospirillum rubrum* occurred in the cytoplasm and then selenium was excreted in the extracellular environment across the plasma membrane and cell wall.

### Tellurite

Two novel strains, *Rhodobacter* sp.: NKPB030619 (Yamada and Matsunaga 1997) and NKPB160041 (Yamada et al. 1997a), were isolated from a marine environment. The strain NKPB030619 removed almost 95% of 0.85 mM of tellurite from the culture medium under photoheterotrophic conditions. Another strain of *Rb. sphaeroides* isolated by Moore and Kaplan (1992) showed resistance to high concentrations of tellurite. All the strains were suggested to have efficiency of tellurite removal from marine water, ponds, lake, and farm drainage.

### Mercury

Contamination of soil, sediment, and water by Hg is a global concern due to its severe toxicity, persistence in the environment, and bioaccumulation potential. Bacterial bioremediation has been regarded as one of the efficient techniques for removal of Hg from contaminated environments (Mahbub et al. 2017). To date, only a few Hg-resistant PNSB have been described. Mukkata et al. (2015) isolated six Hg-resistant strains of PNSB from contaminated shrimp ponds. Among them, *Afifella marina* SSS2-1, *A. marina* SSW15-1, and *Rhodovulum sulfidophilum* SRW1-5 efficiently reduced Hg^2+^ to volatile elemental mercury (Hg^0^) by the enzyme, mercuric reductase. The activity of this enzyme was found under both microaerobic-light and aerobic-dark conditions; however, better activity was observed under aerobic-dark conditions. In addition to the biovolatilization potential, Mukkata et al. (2019) demonstrated that both live and dead cells of the Hg-resistant PNSB strains could also remove Hg^2+^ from aqueous solution by acting as biosorbent. Interestingly, dead cells of the strains were more effective in Hg^2+^ biosorption compared to their live cells.

Apart from enzymatic reduction, a completely different mechanism of Hg^2+^ biovolatilization was described by Grégoire and Poulain (2016). The authors demonstrated that, Hg^2+^ could act as an electron sink resulting in the production of less toxic Hg^0^ and thereby maintain cellular redox homeostasis in photoheterotrophically grown cells of model PNSB strains *Rb. capsulatus, Rb. sphaeroides* and *R. palustris*. Biomethylation by anaerobic microorganisms is a major route of the formation of more toxic organic mercury, particularly monomethylmercury (MeHg), in anaerobic environments (Ma et al. 2019). The utilization of Hg^2+^ as an electron sink during photoheterotrophic growth of PNSB would reduce the rate of formation of more toxic MeHg in contaminated anaerobic-light environments, as exists in shallow sediments. In contrast, the ability of PNSB in biotransformation of Hg^2+^ in anaerobic and under permanent dark sediments remains unknown.

### Other heavy metals

Zinc (Zn) and copper (Cu) are essential micronutrients for plants, animals, and humans. Although relatively non-toxic than other heavy metals discussed in the previous sections, exposure to excessive amounts of these metals can lead to serious health problems (Gaetke and Chow 2003; Plum et al. 2010). Both Zn^2+^ and Cu^2+^ have been found in elevated concentrations in waterbodies and anaerobic sediments (Wang et al. 2011; Yang et al. 2012). PNSB could be promising players in the removal of excess concentrations of these metals from anaerobic or aerobic environments. Few PNSB strains have so far been described for their ability to remove Zn^2+^ and Cu^2+^. For instance, wild-type strain B10 of *Rb. capsulatus* was reported to remove Zn^2+^ by biosorption more efficiently from polluted environments compared to strain RC220 that lacked the resistance conferring endogenous plasmid (Magnin et al. 2014). Panwichian et al. (2010a) isolated two salt tolerant (3% NaCl) PNSB strains, *Rhodobium marinum* strain NW16 and *Rb. sphaeroides* strain KMS24, from shrimp ponds water and sediment, respectively, that could remove both Zn^2+^ and Cu^2+^. Both the strains could remove the heavy metals mainly by complexation with EPS and, to a lesser extent, by bioaccumulation under both microaerobic-light or aerobic-dark conditions (Panwichian et al. 2011). Besides the heavy metals and metalloids discussed above, few studies reported that certain PNSB strains could potentially remove radioactive uranium (Llorens et al. 2012) and caesium (Sasaki et al. 2012a, b) from contaminated soil, water and sediments.

**Fig. 4:**
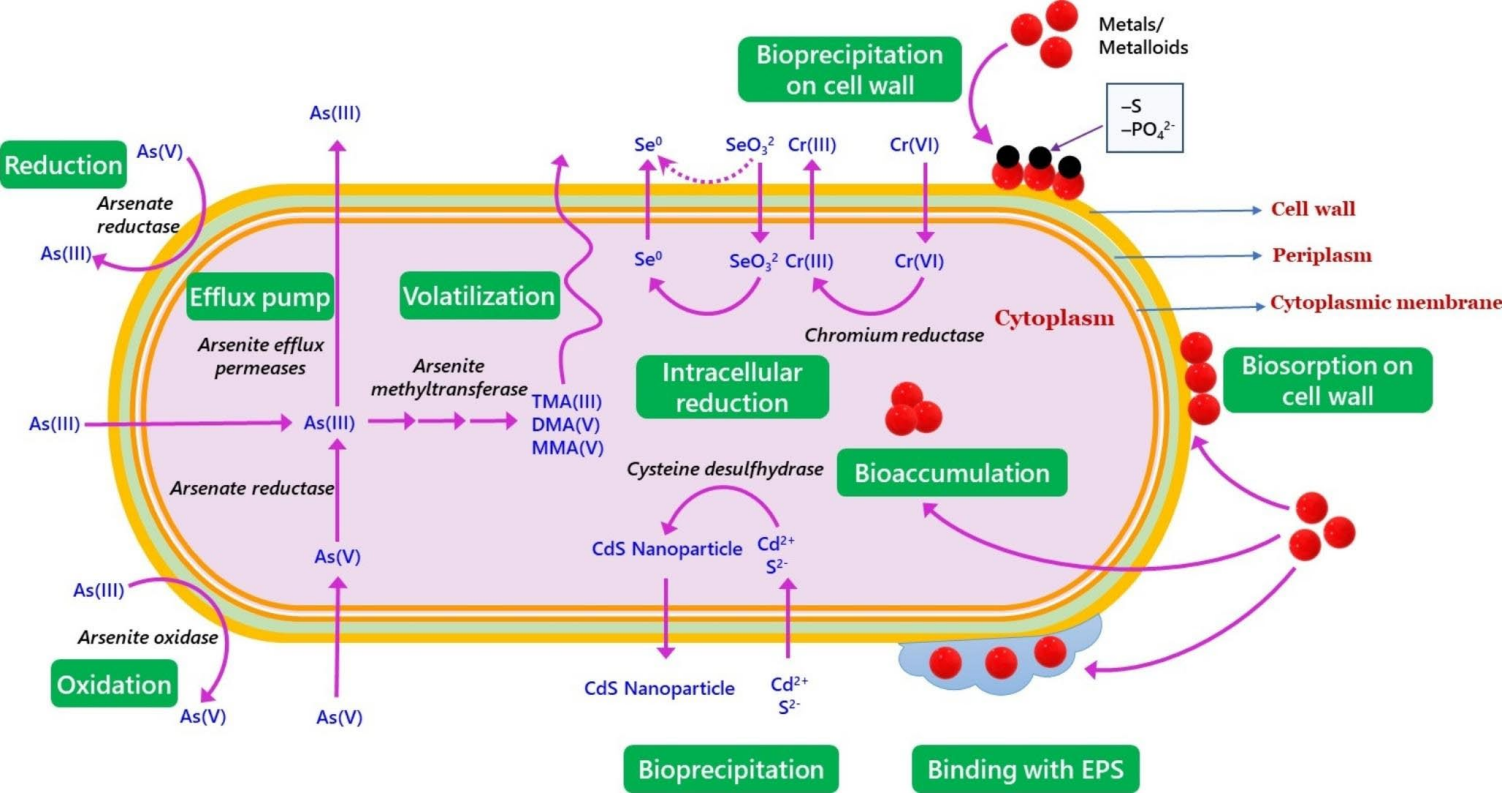
Mechanisms of heavy metal and metalloid resistance in PNSB. They employ diverse mechanisms, including biosorption on cell surface, redox transformation, bioaccumulation, biomethylation and volatilization, and bioprecipitation as metal-phosphates, metal-sulfates and metal-sulfides

Overall, it can be concluded that PNSB resistance to heavy metals and metalloids are prevalent in nature. The mechanisms of metal resistance in PNSB can be broadly categorized into biosorption, precipitation across and on cell walls as metal-sulfide, -phosphate or -sulfate, redox transformation, bioaccumulation, biomethylation, and volatilization of less toxic species like Hg^0^ and TMA(III) (Fig. 4). While certain studies have already shed some light on the mechanisms of PNSB toward resistance to heavy metals and metalloids, knowledge of associated genes, enzymes, pathways, and gene regulations is yet to be generated.

### Biodegradation of hazardous organic pollutants by PNSB

PNSB have been well known for their ability to utilize a wide range of organic compounds mainly under photoheterotrophic conditions. Certain strains of *Rhodopseudomonas palustris* can utilize a variety of organic compounds, including benzoate, hydroxylated- and methoxylated aromatic acids, chlorobenzoates, polychlorinated biphenyls, dinitrophenols, phenolic compounds, aromatic aldehydes, hydroaromatic acids, several plant-derived lignocellulosic compounds, and certain xenobiotic compounds (Harwood and Gibson 1988; Kamal and Wyndham 1990; Khanna et al. 1992; Montgomery and Vogel 1992; McGrath and Harfoot 1997; Austin et al. 2015). Much of the information on biochemical mechanisms, genes, and regulations of anaerobic benzoate degradation was revealed from the investigations with *R. palustris* (Harwood and Gibson 1986; Geissler et al. 1988a, b; Kim and Harwood 1991; Egland et al. 1995, 1997, 2001; Harrison and Harwood 2005; Hirakawa et al. 2015). *R. palustris* CGA009 has also been the model organism for studies of anaerobic degradation of lignin-derived aromatic acids (Harwood and Gibson 1988; Austin et al. 2015). In addition to the known mechanisms of anaerobic biodegradation, the genome sequence of the metabolically versatile bacterium also revealed the presence of an extended range of degradation genes previously unknown (Larimer et al. 2004). Although anaerobic biodegradation of several substituted aromatic compounds has been established, the involvement of PNSB in the biodegradation of unsubstituted aromatic compounds, such as benzene and polycyclic aromatic hydrocarbons (PAHs) remains unknown.

Halogenated hydrocarbons are widespread toxicants and persistent environmental pollutants released into the environment because of their use in agriculture and various industrial processes. Several aerobic and anaerobic bacteria have been described for their ability to detoxify the chemicals from contaminated environments. Under anaerobic-light conditions, *R. palustris* strain WS17 could carry out reductive dehalogenation and complete mineralization of 3-chlorobenzoate (3-CBA) in the presence of benzoate as the co-substrate (Kamal and Wyndham 1990). The strain was unable to degrade 3-CBA under aerobic conditions or in the absence of benzoate under phototrophic conditions. Similarly, three strains of *R. palustris* isolated by Oda et al. (2001) initially required benzoate for 3-CBA degradation. Interestingly, after 1–3 months of incubation in the presence of both benzoate and 3-CBA, the strain developed the ability to grow solely not only on 3-CBA but also in 2- and 4-CBA. In contrast, *R. palustris* strain RCB100 could utilize 3-CBA as the sole carbon source (Egland et al. 2001). Similarly, *R. palustris* strain DCP3 reductively dehalogenated and utilized 3-CBA or 3-bromobenzoate as the sole carbon source under photoheterotrophic conditions (Van der Woude et al. 1994). Oxygen level in the medium had a significant effect on 3-CBA degradation ability of the strain DCP3 (Krooneman et al. 1999). Biodegradation of 3-CBA was inhibited in the presence of a low level (3 µM) of O_2_ and only occurred when an additional carbon source was supplied. These observations suggest that 3-CBA degradation in the natural environments by PNSB may occur only in anaerobic-light conditions. Reductive dehalogenation of 2,3,5,6-tetrachlorobiphenyl was reported in a phototrophic enrichment culture with acetate as the carbon source (Montgomery and Vogel 1992). The first phototrophic degradation of an aromatic compound was demonstrated for toluene in *Blastochloris sulfoviridis* strain ToP1 (Zengler et al. 1999). Nearly complete utilization of toluene was observed only in the presence of light, suggesting the light-dependent nature of the substrate degradation process. This study also demonstrated that up to 1% photoheterotrophic bacterial population of sediment and sludge samples cultivable with acetate could grow on toluene. Therefore, toluene-degrading photoheterotrophic PNSB may be widespread in aquatic habitats and play a crucial role in natural attenuation.

Photoheterotrophic degradation of non-aromatic organic pollutants has been described in several strains of *R. palustris*. Tributyl phosphate, a toxic organophosphorus compound commonly released from nuclear fuel processing and chemical industries, was degraded by *R. palustris* (Berne et al. 2007). *R. palustris* strain RP2 could degrade diesel fuel comprising C9-C36 hydrocarbons (Venkidusamy and Megharaj 2016). This electrogenic bacterium was capable of direct electrode respiration and electron transport through electrically conductive nanofilaments; these properties can be exploited in developing bio-electrochemical remediation systems. Acrylamide degradation by *R. palustris* strain Ac1 has been the only report demonstrating anaerobic photoheterotrophic utilization of the neurotoxin (Wampler and Ensign 2005). Acrylamide degradation by this bacterium proceeds via the formation of acrylate, which is further metabolized to propionate. The involvement of PNSB in the biodegradation of pesticides has been suggested. For instance, the biodegradation of a pyrethroid pesticide, fenpropathrin, by *Rhodopseudomonas* sp. strain PSB07-21 was reported (Luo et al. 2019). Zhang et al. (2012) claimed that *Rhodobacter sphaeroides* W16 could degrade atrazine. Although biodegradation of several other pyrethroid and organophosphate pesticides by certain members of PNSB has been suggested, conclusive evidence showing such pesticide degradation is lacking.

Most of the studies with organic pollutant degradation by PNSB were conducted in photoheterotrophic conditions. However, PNSB in their typical habitats, for instance, in shallow lakes, lagoons, and agricultural fields, experience oxic-anoxic switch. In such conditions, organic compounds degradation by PNSB may involve the participation of both aerobic and anaerobic metabolism for complete degradation. Evidence of this hypothesis was demonstrated in phenol degradation by *Rhodopseudomonas palustris* PL1 (Noh et al. 2002). The organism could transform phenol to 4-hydroxyphenylacetate under phototrophic conditions in the presence of an additional growth substrate, either acetate, malate, benzoate, or cinnamate as growth substrate. However, further metabolism of 4-hydroxyphenylacetate required the presence of a low concentration of oxygen. This requirement suggested the necessity of aerobic enzymes for aromatic ring cleavage *via* either the homogentisate or homoprotocatechuate pathways. Interestingly, the genome of *R. palustris* CGA009 has genes for aerobic aromatic compound degradation *via* oxygenase-dependent ring cleavage pathways (Larimer et al. 2004). Thus, the combination of aerobic-anaerobic metabolism of organic pollutants may thus lead to the development of efficient bioremediation techniques. PNSB can also perform aerobic metabolism of several organic compounds (Harwood and Gibson 1988). A recent study suggested that *Rhodopseudomonas palustris* strain YSC3 could aerobically degrade the brominated flame retardant hexabromocyclododecane (HBCD). Thus, future investigations should consider examining the biodegradation ability of PNSB under aerobic, anaerobic, and a combination of aerobic-anaerobic conditions. Organic compounds that did not support the growth of PNSB can be detoxified via a photobiological transformation process. For instance, aniline did not support the photoheterotrophic or chemoheterotrophic growth of *Rb. sphaeroides* OU5 as a sole carbon or nitrogen source, although aniline was transformed to indole derivatives in the presence of light (Shanker et al. 2006).

A recent development in the PNSB-based bioremediation technology involves the utilization of photosynthetic and electrogenic properties of the bacteria in the construction of photobiological bioreactors and photocatalytic cells for the removal of recalcitrant hazardous organic pollutants from wastewaters. These approaches not only enhance bioremediation efficiency but also contribute to a circular economy by means of the generation of electricity and hydrogen biofuel. For instance, Venkidusamy and Megharaj (2016) isolated *R. palustris* strain RP2 from the anodic biofilms of hydrocarbon-fed microbial electrochemical remediation system, which degraded 47% of 800 mg L^–1^ diesel fuel with concomitant production of electricity under photoheterotrophic conditions. Sogani et al. (2021) constructed a photo-assisted microbial fuel cell (MFC) consisting of *R. palustris* for the degradation of estrogenic chemical, ethinylestradiol (EE2), under anaerobic-light conditions. The exoelectrogenic activity of *R. palustris* biofilm in the photo-assisted MFC resulted in the removal of 89.82% EE2 from an initial concentration of 1 mg L^–1^. Addition of EE2 to glycerol co-substrate in the MFC also enhanced hydrogen generation by 63%.

The integration of photocatalysis and biodegradation has long been utilized in wastewater treatment technology for the removal of recalcitrant organic compounds. When applied independently, photocatalysis could lead to the accumulation of reactive oxygen species and toxic photodegradation products, for example, toxic quinones from PAHs (Bertilsson and Widenfalk 2002). Similarly, many hazardous organic pollutants can be quite resistant to microbial degradation. Intimately coupling of photocatalysis and biodegradation (ICPB) technique harnesses the power of photocatalysis and simultaneous microbial biodegradation of photodegradation products while preventing the accumulation of toxic by-products and leading to complete mineralization. Zhang et al. (2017) constructed an ICPB system with photocatalyst (g-C_3_N_4_-P_25_) and calcium alginate encapsulated *Rhodospirillum* sp. to remove reactive brilliant red X-3b dye. Almost 94% of 50 mg L^–1^ of the dye was removed by the ICPB system. Liu et al. (2022) constructed an ICPB system by coupling *R. palustris* immobilized in sodium alginate to carbon nanotube-silver modified titanium dioxide photocatalytic composite (CNT-Ag-TiO2, CAT) for the removal of a recalcitrant azo dye, Congo red, from wastewater. In the presence of glucose as the co-substrate, the sodium alginate trapped cells alone could only transform a small amount of Congo red into aromatic intermediates. CAT could transform the dye into long-chain alkanes and a few aromatic hydrocarbon compounds. However, in the ICPB system, Congo red was first degraded into long-chain alkanes by the superoxide and hydroxyl radicals of CAT product and then completely mineralized by *R. palustris*, resulting in enhanced removal with reduced accumulation of intermediates. Similarly, Liu et al. (2023) demonstrated efficient removal of azo dyes from wastewater by a composite constructed with S-scheme heterojunctions photocatalyst g-C_3_N_4_/MoS_2_ coupled to *R. palustris* with chitosan-modified polyurethane sponge as a carrier. The composite system achieved 99.50, 97.50, and 99.50% degradation of Congo red, methyl orange, and carmine, respectively. It has been proposed that the dyes adsorbed on the carrier material were first oxidized by the actions of the strong oxidizing radicals produced by the photocatalyst to alkanes which were subsequently mineralized by the bacterium. Therefore, the ICPB approach employing PNSB as the biological agent can be an effective remediation technology for the removal of hazardous organic pollutants that would generally be recalcitrant to microbial degradation alone. At the same time, such technique could also be invaluable in mineralization of toxic photodegradation products and thereby could lead to efficient and complete removal of organic pollutants.

### Prospects and recommendations

A critical perusal of the literature describing the bioremediation potentials of toxic environmental pollutants by PNSB reveals some crucial facts. First, PNSB-based bioremediation has not received due attention from researchers, as indicated by the limited number of available reports. Second, the scope of many such studies was confined to laboratory-scale investigations lacking observations from field-scale experiments. Third, the impact of biotic and abiotic factors that could affect the performance of PNSB in pollutant bioremediation has rarely been addressed. Fourth, current knowledge on the ability of PNSB to degrade hazardous aliphatic and aromatic organic compounds, especially unsubstituted compounds such as PAHs, is severely limited. Fifth, the molecular genetics and biochemical processes of the detoxification mechanisms are largely unknown.

**Fig. 5:**
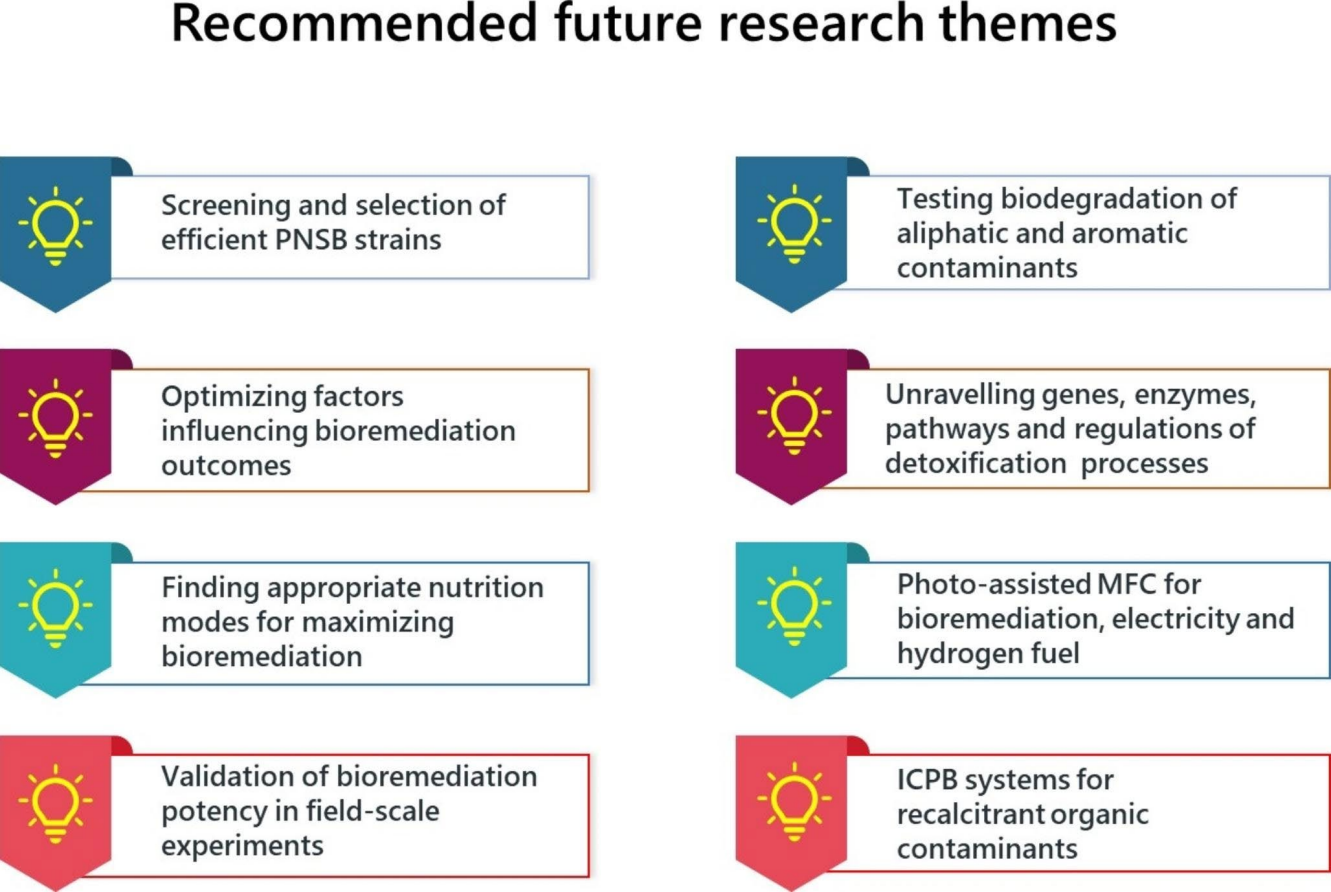
Recommended future research directions for the development in the field of PNSB-based bioremediation of hazardous heavy metals, metalloids, and organic pollutants

The current state of understanding is inadequate for evaluating the suitability of PNSB in the bioremediation of environmental pollutants. Figure 5 presents major areas of future research that should receive significant attention. Isolation of efficient bacteria from contaminated environments is a prerequisite to the development of a successful bioremediation strategy. Significant research should be directed toward the isolation of novel PNSB capable of heavy metal detoxification and organic pollutants biodegradation. Before searching for an efficient PNSB strain, it seems necessary to evaluate whether the site is conducive to the growth and proliferation of PNSB since most members prefer to grow in well-illuminated microaerobic or anaerobic conditions. A screening program should also emphasize the selection of strains that are resistant to several biotic and abiotic factors. Particularly, strains that can perform in a range of temperatures, pH, and salinity are better candidates for bioremediation. Additionally, the ability to withstand the toxicity of co-occurring chemicals in the contaminated sites should also be considered in the selection criteria. Instead of focusing only on the photoheterotrophic process, the ability to detoxify under aerobic and dark conditions may help recover some efficient PSNB strains with bioremediation potentials. Laboratory findings may greatly vary from what could be observed in a real environment. Therefore, the actual potential of PNSB should be appreciated based on their performance in contaminated sites. In addition to screening effective strains and evaluating the bioremediation efficiency, the molecular mechanism of detoxification should be investigated in detail. Since photoheterotrophic utilization depends on the anaerobic-light conditions and many aromatic compounds are prone to photodegradation, particular attention should be paid when investigating photodegradable organic compounds, such as PAHs. Otherwise, most of the chemicals would be degraded upon induction with photon energy, leaving little substrate for biodegrading bacteria, and the photodegradation product could be lethal. Additionally, the degradability of toxic photodegradation products, such as quinones, should be investigated. The power of next-generation “omics” technology and system biology should be exploited in understanding the molecular mechanism of the bioremediation process and deciphering unknown potentials of PNSB. Coupling the biodegradation ability of PNSB with chemical or photocatalytic systems may also be effective in removing recalcitrant and toxic organic compounds.

## Data Availability

No new data were created or analyzed in this study.
